# Are Movements Necessary for the Sense of Body Ownership? Evidence from the Rubber Hand Illusion in Pure Hemiplegic Patients

**DOI:** 10.1371/journal.pone.0117155

**Published:** 2015-03-16

**Authors:** Dalila Burin, Alessandro Livelli, Francesca Garbarini, Carlotta Fossataro, Alessia Folegatti, Patrizia Gindri, Lorenzo Pia

**Affiliations:** 1 SAMBA (SpAtial, Motor & Bodily Awareness) Research Group, Psychology Department, University of Turin, Turin, Italy; 2 NIT Neuroscience Institute of Turin, Turin, Italy; 3 San Camillo Hospital, Turin, Italy; Duke University, UNITED STATES

## Abstract

A question still debated within cognitive neuroscience is whether signals present during actions significantly contribute to the emergence of human’s body ownership. In the present study, we aimed at answer this question by means of a neuropsychological approach. We administered the classical rubber hand illusion paradigm to a group of healthy participants and to a group of neurological patients affected by a complete left upper limb hemiplegia, but without any propriceptive/tactile deficits. The illusion strength was measured both subjectively (i.e., by a self-report questionnaire) and behaviorally (i.e., the location of one’s own hand is shifted towards the rubber hand). We aimed at examining whether, and to which extent, an enduring absence of movements related signals affects body ownership. Our results showed that patients displayed, respect to healthy participants, stronger illusory effects when the left (affected) hand was stimulated and no effects when the right (unaffected) hand was stimulated. In other words, hemiplegics had a weaker/more flexible sense of body ownership for the affected hand, but an enhanced/more rigid one for the healthy hand. Possible interpretations of such asymmetrical distribution of body ownership, as well as limits of our results, are discussed. Broadly speaking, our findings suggest that the alteration of the normal flow of signals present during movements impacts on human’s body ownership. This in turn, means that movements have a role *per se* in developing and maintaining a coherent body ownership.

## Introduction

Body ownership is the conscious experience of the body as one’s own [1]. Indeed, it is an ubiquitous perceptual experience that stands at the root of human nature since we all sense what it’s like having a body and we experience the boundaries between our own body and the external world [2].

Recent theoretical and methodological advances have leaded to the development of new approaches to examine in depth the neurocognitive processes underpinning the conscious experience of one’s own body. Perhaps, one of the most compelling demonstration of the mechanisms subserving body ownership has been obtained in healthy participants by means of an experimental manipulation in which the physical constraints subserving body ownership are altered. Such paradigm is known as the ‘rubber hand illusion’ [3]. Basically, it is shown that synchronous, but not asynchronous, touches onto a static visible rubber hand and onto the static hidden participants’ hand produce the compelling change in the believes of ownership of that hand (e.g., [3–7]). The attribution is typically measured both objectively (i.e., the perceived location of one’s own hand toward the rubber hand) and subjectively (the experience of owning the rubber hand). It is worth noticing that incongruent rubber hand postures, incongruent identity (e.g., neutral objects) does not seem to induce the illusion (e.g., [8]). The rubber hand illusion effects are explained with the fact that when the rubber hand is congruent with the participant’s hand in terms of posture and identity, the conflict between somatosensory representations of the own hand and vision of the fake hand disappears in favor of a strong multisensory integration (i.e., touch, proprioception and vision). This, in turns, induces a unitary multisensory perception of the fake hand as one’s own hand receiving the tactile stimuli [9,10]. Interestingly, other recent approaches have extended this paradigm to the whole body by employing virtual reality [11,12].

The classical version of the rubber hand illusion paradigm [9,10] reveals that when visual and tactile stimuli delivered to one‘s own body part match in terms of space, time and identity, a feeling of ownership arises. However, human body receives stimulations also during actions and, in fact, the feeling that one's body belongs to oneself is present also when we move: “I know that this moving hand is mine”. In these situations, further signals add to vision and touch: skin/joint receptors, muscles spindles give us kinesthetic information (see [13] for a review). Additionally, during willed actions the brain process also centrally generated motor commands (efferent signals) and the sensory predictions they produce (efference copy; see, for instance [14]). Consistently with these observations, recent studies aimed at examining whether or not these other signals are as important as tactile ones in terms of body ownership development [15–21]. Broadly speaking, most of those studies modified the original rubber hand paradigm comparing active and passive movements conditions with the static version. Overall, this literature presents conflicting results, that is some papers provided hints that body ownership increases during movements [15,19,22], others that it decreases [20], and some others reported no differences between movement and no-movement conditions [16,21].

Overall, at present there is no consensus on if, and to which extent, movements contribute to the emergence of body ownership. In the present study, we aimed at answer this question within a neuropsychological perspective. We selected patients affected by a pure form of left hemiplegia, that is complete paresis of the left upper limb but no proprioceptive and/or tactile deficits. In other words, these patients had been keeping to receive signals in static (touch and vision) but not in dynamic (kinesthetic, efferent and efference copy) conditions. If movements are necessary for the construction of body ownership, we predicted in patients, respect to healthy subjects, 1) stronger rubber hand illusion effects for the left affected hand, and 2) similar illusory effects on the right unaffected hand. In other words, the hemiplegic hand would display a weaker or more flexible sense of body ownership, whereas the right unaffected hand the same body ownership.

## Materials and Methods

### Participants

We retrospectively selected the participants of the study from a large sample of stroke patients with right hemispheric lesions (documented by computerized tomography) and no history of substance abuse/previous neurological diseases, admitted to different rehabilitation centers. The prerequisite to be included in this study was the presence of a complete left upper limb hemiplegia and no tactile loss (in order to administer the rubber hand illusion paradigm). Nonetheless, in order to focus entirely on the contribution of movements to body ownership, we excluded all patients affected by propriceptive deficits, personal/extrapersonal neglect and anosognosia for hemiplegia [23,24]. Eight right-handed patients (six men; mean age 63.6 years, SD = 11.4 years; mean educational level 9.2 years, SD = 4 years; hereinafter HP group) and seventeen (three subjects form the original samples of twenty were excluded as outliers) age and educational level-matched right-handed healthy subjects (nine men; mean age 66.1 years, SD = 8 years; mean educational level 9.5 years, SD = 4.6 years; hereinafter C group) participated in the study after having given written informed consent according to the declaration of Helsinki. The study was approved by the ethic committee of the University of Turin (Project “Conscious brain: neural basis of motor and body awareness”, prot. 1/2014/B1).

### Neurological and neuropsychological assessment

Handedness was assessed with the Edinburg inventory [25], whereas patients’ screening for global cognitive functioning was evaluated with the Italian version of the Mini Mental State Examination [26]. Contralesional motor, tactile and proprioceptive defects as well as unawareness for motor deficits were assessed according to a standardized protocol [27,28] in which scores range from 0 (no deficit) to 3 (severe deficit), whereas those for unawareness of hemiplegia ranges from 0 (no deficit) to 2 (sever deficit). The presence of left extrapersonal neglect was assessed with line bisection task [29] and a Diller cancellation [30] tasks, whereas the presence of personal neglect was assessed with the Fluff test [31]. Patients’ demographic, clinical and neuropsychological data are reported in Table 1.

**Table 1:** Demographical, neurological and neuropsychological data of the HP group.

Id	Sex	Age	Edu (y)	Ons (days)	Aet	Lesion	N.E.			AHP	MMSE	Line bisection	Diller	Fluff
							M	T	P					
BM	F	78	5	72	I	Ic	3	0	0	0	26	8/9	2	0
BP	M	77	17	69	I	T, BG	3	0	0	0	28	8/9	2	0
CD	M	64	8	46	I	F, P	3	0	0	0	27	9/9	−1	0
MG	M	63	8	258	I	Bg	3	0	0	0	27	9/9	0	0
PA	M	62	5	76	H	Ic	3	0	0	0	28	9/9	0	0
PL	M	69	10	91	I	F, T, P	3	0	0	0	26	8/9	2	0
PF	M	51	8	53	I	Bg	3	0	0	0	27	9/9	0	0
ZE	F	45	13	120	H	F	3	0	0	0	25	9/9	0	0

Id = patients' Identification number. Sex: M = Male, F = Female. Edu: years (y) of formal education. Aet: Aetiology, H = hemorrhage, I = ischemia. Lesion: F = frontal, T = temporal, P = parietal, Bg = basal ganglia, Ic = Internal capsule. Ons = Illness onset. N.E = Neurological examination, Contralesional Motor (M), Tactile (T), and Proprioceptive (P) neurological deficits (the two values refer to the upper and lower limb respectively); scores ranged from normal (0) to severe defects (3). AHP = Unawareness of hemiplegia (the two values refer to the upper and lower limb, respectively); scores ranged from normal (0) to severe defects (2). MMSE: Mini-Mental State Exam score (0–30, cut off 24). Line bisection: number of correct bisections (0/9–9/9, the Behavioural Inattention Test). Diller: left minus right omitted targets (0–52, cut off > 3 [54]). Fluff. Scores ranged from normal (0) to severe defects (3).

### Experimental settings and procedures

**Fig 1:**
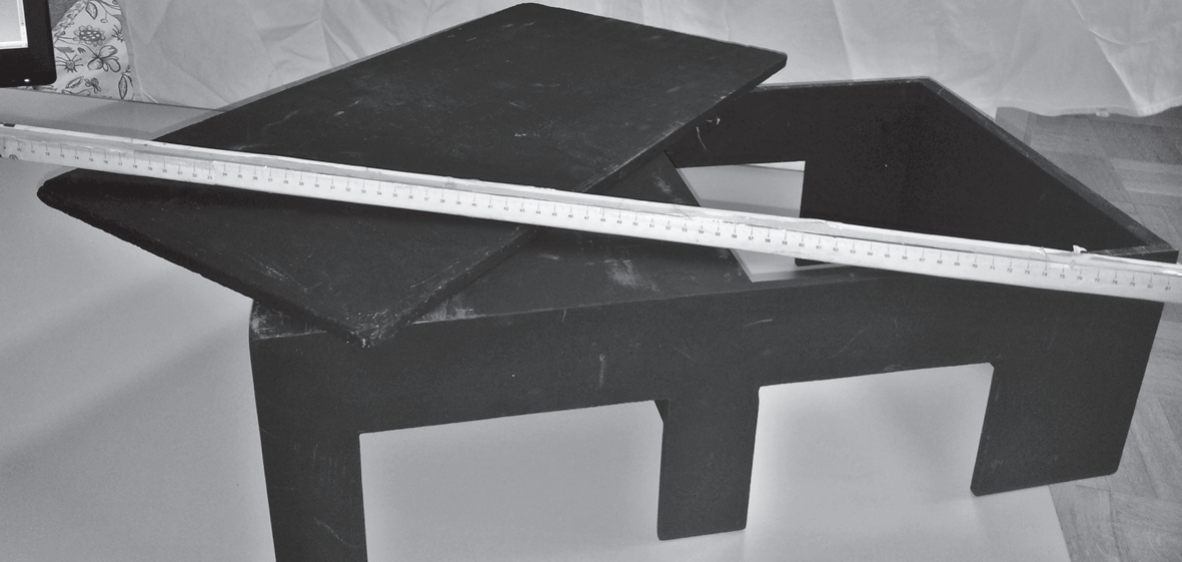
Picture of the materials employed in the experiment.

We employed a black wooden box (60 cm x 40 cm x 20 cm) divided in two equal parts (30 cm x 30 cm x 20 cm) by a perpendicular panel. One of the two parts was open to the view. Two square holes (12 cm x 12 cm) on either the horizontal sides of the box allowed placing both the participant’s arm and the rubber hand (left or right). The set up included also an automotive wooden panel (30 x 40) and a wooden stick (100 cm long) on which was previously applied a tailor-ruler (0 to 100 cm). See Fig. 1.

The box was placed 15 cm in front of the participant’s torso and arranged to have the rubber hand (left or right) aligned with the correspondent participants’ shoulder (left or right). Participants were familiarized with the setting, procedures and all rating scales. Then, the experimenter placed the participants’ arm (left or right) in a fixed location within the part of the box hidden to the view. Fingers were pointing forward and palm was facing down. Then, the rubber hand (left or right) was placed in the other half of the box (open to the view) parallel and in the correspondence of the participants’ shoulder. The distance between the real and the rubber hand was approximately 25 cm.

As first, the experimenter sat in front of the participant and placed the automotive panel on the open part of the box in order to cover also the rubber hand. Then, the experimenter placed on the top of the wooden box the stick. The participant had to report the number correspondent to the position of their index finger (six trials), which was referred as the proprioceptive judgement. In order to avoid number repetitions, the position of the stick was randomly varied across trials.

**Fig 2:**
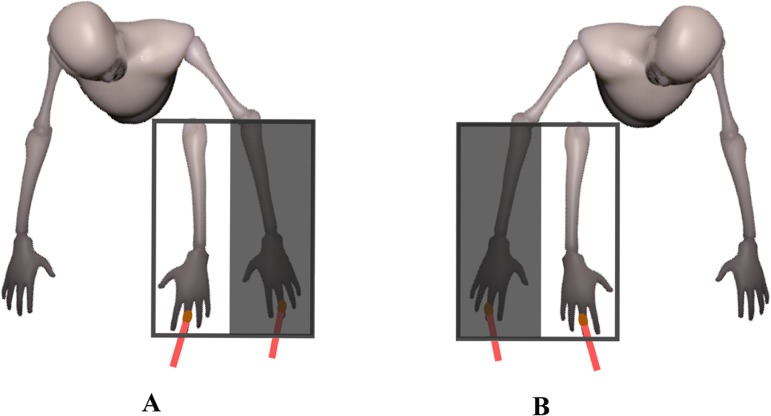
Picture of the experimental set-up. Left hand stimulation (Fig. 1A) and right hand stimulation (Fig. 1B).

Secondly, the experimenter removed the automotive panel and asked to participants to always look to the index fake finger during the subsequent stimulations. Then, the experiment started to stroke both the participants’ index finger and the rubber hand index finger with two equal small brushes for 180 sec. In the synchronous condition, the two hands (left or right) were stimulated simultaneously (one trial for each hand), whereas in the asynchronous condition, the stimulations were temporally incongruent (one trial for each hand). See Fig. 2.

Thirdly, after each kind of stimulation, participants were asked to indicate the position of their index finger on the ruler after the experimenter covered the rubber hand with the automotive panel (i.e., proprioceptive judgment) and to fill out a questionnaire about the experience of the illusion [3]. The questionnaire was composed of six questions (see appendix), three (Q1–Q3) to capture different aspects of the illusory perception (e.g., the sensation of touches on the rubber hand and the change in the believes of ownership of that hand), whereas three (Q4–Q6) served as control questions for task compliance and susceptibility effects. Participants had to rate their agreement/disagreement on a seven point Likert scale with a range from “+3” (agree very strongly) to “−3” (disagree very strongly) where “0″ corresponded to neither agreeing nor disagreeing.

In order to avoid any possible carry-over effects of the illusion, after each post stimulation trial, participants had 60 sec of rest. Then, another pre-stimulation session preceded the next post-stimulation trial.

In summary, there were four conditions randomized between subjects: left hand synchronous stimulation, left hand asynchronous stimulation, right hand synchronous stimulation, right hand asynchronous stimulation. The order of presentation of these four conditions was randomized by balancing the order of the stimulated hand (left, right) and the order of stimulation (synchronous or asynchronous) separately across subjects.

### Statistical analysis

A preliminary analysis compared (t-test for independent samples by group) between groups the pre stimulation proprioceptive judgment. Then, the pre stimulation values were subtracted from the post stimulation values and referred as the proprioceptive drift [8,32]. Positive values represented a mislocalization towards the rubber hand). The normality of the distribution of the propriceptive drift values was evaluated by a Kolmogorov-Smirnov test, whereas the homogeneity of variance by means of the Brown-Forsythe-test particularly useful for unequal sample sizes. According to these latter analysis, the proprioceptive drift was analyzed by means of a repeated measure’s ANOVA with GROUP (HP, C) as between subjects factor, STIMULATION (synchronous, asynchronous) and HAND (left, right) as within subjects factors. Significant level was set at p < .05, when a significant interaction was detected; post-hoc analysis were conducted with Duncan test. Since with small sample sizes, the lack of significance might be due to insufficient power, when required we performed retrospective power analysis (alpha level = .05) in the HP group.

The subjective rating for each of the six questions was standardized by means of an ipsatization procedure (see [33] details) useful to neutralize any bias of response). Then, the subjective rating for each question was analyzed by means of a repeated measure’s ANOVA with GROUP (HP vs. C) as between subjects factor, STIMULATION (synchronous, asynchronous) and HAND (left, right) as within subjects factor. Significant level was set at p < .05, when a significant interaction was detected; post-hoc analysis were conducted with Duncan test. When required we performed retrospective power analysis (alpha level = .05) in the HP group.

## Results

### Proprioceptive drift

Pre stimulation proprioceptive judgment (p > .05) did not differ between groups (synchronous left: C mean = 2.28, SD = 2.83; HP .29, 3.46. Asynchronous left: C 2.25, 3; HP 1.19, 3.27. Synchronous right: C 1.06, 2.98; HP 1.92, 2.39. Asynchronous right: C 1.25, 2.83, HP 1.7, 2.14).

**Fig 3:**
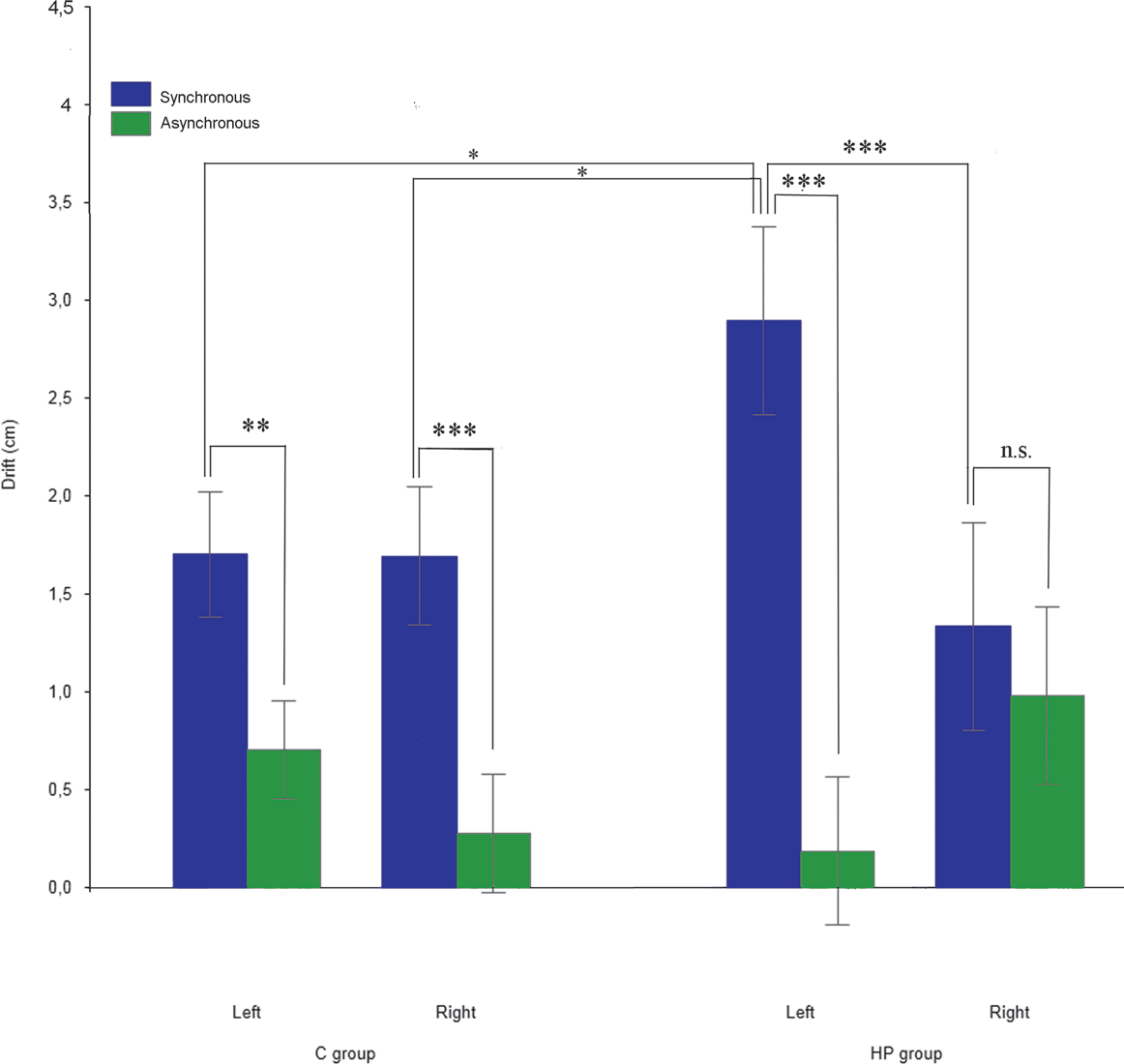
Graphic representation of the proprioceptive drift analysis on the two groups. Error bars represent standard errors. Asterisks indicate significant comparisons (* p < 0.05** p < 0.01*** p < 0.005).

Since data were distributed normally (p > .05) and variances were homogenous (p > .1) between groups, we performed the 2x2x2 repeated measure’s ANOVA on the proprioceptive drift (cm). The main factor STIMULATION was significant [F (1,24) = 28.72, p < .005] with the drift being higher in the synchronous (mean = 1.97 cm, SE = .22 cm) respect to the asynchronous (mean = .54 cm, SE = .28 cm) condition. However, the significance of the STIMULATION x HAND x GROUP interaction [F (1,24) = 18.95, p < .001] revealed that in the HP group such difference was present (post hoc, p < .005) only when the left affected hand was stimulated (synchronous: 2.9, ± .48; asynchronous: .19 ±.38). Indeed, the drift in the synchronous condition was higher (post hoc, p < .05) respect to when C group’s hands were stimulated synchronously (left: 1.7, ± .32; right: 1.69, ± = .35), and HP group’s right unaffected hand was stimulated synchronously (1.33, ± .53) or asynchronously (.98, ± = .46). On the contrary, when the right hand was stimulated, the higher drift (post hoc, p < .005) in the synchronous respect the asynchronous condition was present only in the C group (synchronous: 1.69, ± .35; asynchronous: .28, ± .3). The between groups comparisons within each condition (i.e. synchronous vs synchronous and asynchronous vs asynchronous) were not significant (post hoc, p > .05). The retrospective power analysis (alpha level = .05) in the HP group when the right hand was stimulated resulted in: Power .24, effect size = .51. See Fig. 3.

### Subjective rating

We performed the 2x2x2 repeated measure’s ANOVA on the subjective rating (z-score) for each question separately.

In Q1, the main factor STIMULATION was significant [F (1,24) = 32.63, p < .005] with a positive (presence of the illusion) and higher rating in the synchronous (Z = 1.45, SE = .15) respect to the synchronous (.01, ± = .16) condition. The significance of the STIMULATION x HAND x GROUP interaction [F (1,24) = 18.95, p = .047] revealed that in the HP group such pattern was present (post hoc, p < .005) only when the left affected hand was stimulated (synchronous: .19, ± .29; asynchronous: −.04 ± .32). On the contrary, when the right hand was stimulated, the pattern was present (post hoc, p < .05) only in the C group (synchronous: .15, ± .23; asynchronous: .02 ± .02). The retrospective power analysis (alpha level = .05) in the HP group when the right hand was stimulated resulted in: Power .3, effect size = .36.

Respect to Q2, the main factor STIMULATION was significant [F (1,24) = 26.39, p < .005] with a positive and higher rating in the synchronous (.65, ± .15) respect to the asynchronous (−.3, ± .09) condition.

**Fig 4:**
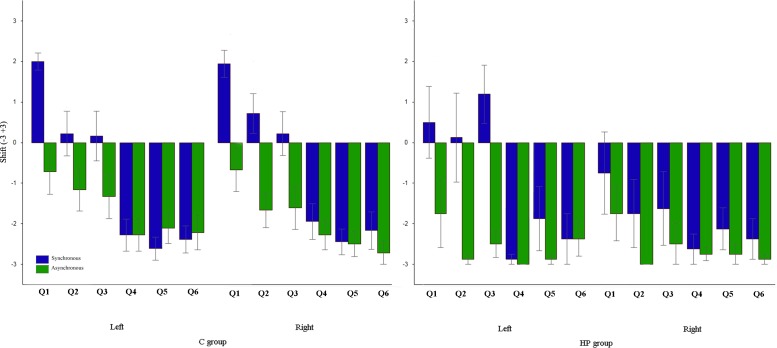
Graphic representation of subjective rating analysis on the two groups. Error bars represent standard errors.

In Q3, the main factor STIMULATION was significant [F (1,24) = 27.95, p < .005] with a positive and higher rating in the synchronous (.86, ± .01) respect to the asynchronous (−.21, ± .14) condition. However, the significance of the STIMULATION x HAND x GROUP interaction [F (1,24) = 18.44, p < .005] revealed that in the HP group such pattern was present (post hoc, p < .005) only when the left affected hand was stimulated (synchronous: 2.09, ± .43; asynchronous: −.19 ± .26). Indeed, the drift in the synchronous condition was higher (post hoc, p < .05) respect to when C group’s hands were stimulated synchronously (left: .61, ± .28; right: .7, ± = .19), and HP group’s right unaffected hand was stimulated synchronously (.03, ± .3) or asynchronously (−.32, ± = .24). On the contrary, when the right hand was stimulated, the pattern was present (post hoc, p < .05) only in the C group (synchronous: .69, ± .2; asynchronous: −.21 ± .17). The retrospective power analysis (alpha level = .05) in the HP group when the right hand was stimulated resulted in: Power .25, effect size = .52). None of the analysis on the other questions resulted to be significant. See Fig. 4.

## Discussion

With the present investigation, we aimed at analyzing whether signals arising from movements (i.e., kinesthetic, efferent and efference copy) affect body ownership. We administered the rubber hand illusion paradigm to a subgroup of neurological patients who, due to their hemiplegia, had not been keeping to receive signals during movements on their plegic hand. Our results show that, respect to healthy participants, the illusory effects were higher when the affected hand was stimulated, but absent when the unaffected hand was stimulated.

As first, in our study we replicated on healthy participants the typical pattern of the rubber hand illusion for both hands [3–7]. Specifically, after synchronous, but not asynchronous stimulation, the perceived position of the stimulated hand resulted to be shifted much closer to the rubber hand. Consistently, only the three statements known to be linked to the subjective experience of the illusion resulted to have a positive value, namely participants had the change in the belief of ownership over the rubber hand. As regards hemiplegic patients, they displayed the same abovementioned pattern when the left affected hand was stimulated. It is worth noting that groups were equally able in recognizing the temporal synchrony and spatial matching between the observed and felt touch on the left hand (no between groups difference as regards Q1 and Q2).

Nonetheless, the effect of synchronous stimulation on the perceived location of the own hand was significantly higher than the one observed in healthy participants (i.e., the left affected hand was significantly more displaced towards the rubber hand). On the contrary, when the right unaffected) hand was stimulated, synchronous and asynchronous stimulation did not differ in terms of perceived position of the stimulated hand and no statements resulted to have a positive value (i.e., patients did not experience the change in the believes of ownership over the rubber hand).

The idea that movements can shape the subjective experience of the body as one’s own is mainly based on the fact that human’s body is the interface between the environment and the phenomenal self [34]. Indeed, willed actions represent the translation from the phenomenal states (desires, goals, intentions) into effects on the external world. In other words, human’s body ownership might also rely on the functional relevance of the physical body [35] and, indeed, human’s body is largely given to us as a source or power for action, namely a range of motor potentialities which defines our world by populating it with bodies and objects we can interact with [36]. As we mentioned above, some studies employing a modified version of the original rubber hand paradigm showed that signals arising during actions crucially contribute to the development of the brain’s sense of body ownership. Specifically, Dummer and colleagues [15] reported that when healthy participants controlled a movement employed to induce the illusion of body ownership over the rubber hand, the illusory effects increased of around 23% respect to when the movements were passive. Similarly, Riemer and coworkers [22] demonstrated that the proprioceptive drift was stronger in actively moving rubber hand illusion respect to the classical version, when tested with a manual pointing procedure. Indeed, the possible link between movements and body ownership has been demonstrated by means of experimental manipulations different from the rubber hand illusion. Romano and coworkers [37], for instance, showed that the observation in a mirror box of the reflected opposite arm elicits involuntary movements. Newport and colleagues [38] reported that synchronous stroking induces not only the embodiment of a fake limb but affects also subsequent motor performance (i.e. reaching error). Rognini and co-workers [39] showed in a virtual reality set up that visuo-tactile integration subserving body ownership is modulated by self-generated movements [39].

Interestingly, Tsakiris and colleagues [19] made a step forward attributing to voluntary actions a specific role in building up body ownership. They demonstrated that the drift in the active movement condition involved both the unstimulated and stimulated fingers, whereas the drift in passive movement condition was strictly localized on the stimulated finger. Hence, the authors claimed that while sensory mechanisms generate a sense of body ownership rooted on fragmented and/or local representation of single body parts, voluntary actions would spread across the whole body inducing a coherent sense of bodily self. In other words, the feeling of unity of bodily self-consciousness would derive from action, and not from sensation, and willed actions would constantly provide precise spatio-temporal signals available to predict proprioceptive and/or visual feedbacks.

The above-mentioned literature suggests that it is predictable that an enduring absence of movements might interrupt the flow between accurate spatio-temporal information and predictions of feedbacks. This, in turn, might affect body ownership *per se*. Accordingly, some recent studies on clinical populations with movement disorders seem to suggest that this might be the case. Patients with focal hand dystonia [40] or spinal cord injury [41,42], for instance, seems to have impairment of body ownership measured with the rubber hand illusion. Interestingly, it has been showed that an altered body ownership can affect voluntary actions: the pathological embodiment of someone else’s arm due to brain damages [23,43,44] can affects the patients’ motor program [45].

Consistently with these observations and with our prediction 1), hemiplegic patients presented stronger rubber hand illusion effects on their left affected hand. We suggest that complete hemiplegia pulls off the movements of the contralesional arm and decreases the number of movement-related signals, progressively disrupting the normal integration between afferent and efferent signals for that arm. This, in turn, weakens body ownership, which causes the hemiplegic hand to be more prone to the illusory effect. It is worth noting that in a previous study [42] on a spinal cord injury patient who still experienced the rubber hand illusion for the deafferented body parts, results were explained as a consequence of a pathological dominance of vision over proprioceptive/somatosensory information *per se*, rather than in terms of absence of movements (see also [46,47] for similar interpretations). Contrary to this study, our patients had a complete preserved proprioceptive and somatosensory functioning. Hence, despite a possible role of vision should be examined in the next future, it seems more likely to explain the results of the comparison between patients and controls in terms the only difference between them, i.e., an enduring absence of movements, rather than in terms of full dominance of vision.

Contrary to prediction 2), however, patients did not show any effect on the right hand. Here we might attempt to speculate on a possible interpretation of this unexpected result. The vast majority of everyday life movements requires (at least) some degree of collaboration between hands and truly unimanual activities are difficult to be found [48]. Such activity is automatic and finely coordinated in both temporal and spatial terms. Hence, stroke-induced unilateral motor deficits force to a regular and repeated overuse of the healthy arm in order to achieve actions. Indeed, sometimes this induces transient abnormalities on that arm [49,50]. Hence, here we put forward the idea that an increasing number of movement-related signals, and the consequent heighten of the normal integration between afferent and efferent signals due to the unaffected arm overuse, would enhance body ownership. This would explain the decrease of the rubber hand illusion effects. Put in other words, such asymmetry of available signals might modulate body ownership in opposite directions, that is weakening it for one arm, enhancing it for the other. However, this second conclusion should be taken very cautiously. As first, our interpretation is motor in nature, but we have not assessed movements. Hence, other possible, more parsimonious, explanations must be discussed. One might argue, for instance, that right arm overuse might have simply induced a motor expertise gain. Similarly, the overuse might have leaded hands asymmetries in positions sense [51]. In both cases, one would have expected different accuracy in detecting hand positions. However, before stroking healthy subjects and hemiplegic patients were equally accurate in reporting their real hand position (both of the left and right hand). Secondly, and perhaps more importantly, the absence of evidence is not evidence of absence, particularly when negative results have low power as in our study. In other words, it is not possible to clearly exclude that also in patients synchronous vs. asynchronous stimulations over the right unaffected hand differed one from the other. It is worth noticing that the main reason of the lack of power of patients’ right hand results is that our analysis is based on a quite small group. It is worth noticing that we have focused only on patients with complete left upper limb hemiplegia but without any kinesthetic/tactile loss. Indeed, these deficits are often associated is difficult to obtain a large sample of patients in a reasonable time.

Future studies should add evidence to the idea of an important role of actions on the development of body ownership and should also assess the specific contributions of kinesthetic, efferent and efference copy information. An interesting possibility to obtain larger groups might be examining other conditions of arm use/disuse as, for instance, long-term arms immobilization (e.g., [52,53]). Interestingly, these approaches would also allow to easily control the impact of time. Indeed a correlation between immobilization time and altered body ownership is expected.

## Supporting Information

S1 Appendix (DOCX)
