# Supplementary material for: Are Movements Necessary for the Sense of Body Ownership? Evidence from the Rubber Hand Illusion in Pure Hemiplegic Patients
Source: PLoS One. 2015 Mar 16;10(3):e0117155. doi: 10.1371/journal.pone.0117155 (PMC4361688; doi:10.1371/journal.pone.0117155)
Supplement: S1 Appendix — (DOCX) [file pone.0117155.s001.docx]

**Appendix**

The items of the questionnaire were as follows:

Q1. It felt as if I was feeling the stroking touch in the location where I saw the rubber hand touched

Q2. It seemed as though the touch I felt was caused by the paintbrush touching the rubber hand.

Q3. I felt as if the rubber hand were my hand

Q4. It felt as if my hand were drifting towards the left/right (towards the rubber hand)

Q5. It seemed as if the touch I was feeling came from somewhere between my own hand and the rubber hand

Q6. It felt as if my hand were turning ‘rubbery’
